# Supplementary material for: Extended gate field-effect-transistor for sensing cortisol stress hormone
Source: Commun Mater. 2021 Jan 19;2(1):10. doi: 10.1038/s43246-020-00114-x (PMC7815575; doi:10.1038/s43246-020-00114-x)
Supplement: Supplementary file 1 — Supplementary Information [file 43246_2020_114_MOESM1_ESM.pdf]

Supplementary information

Extended gate field-effect-transistor for sensing cortisol stress hormone

Shokoofeh Sheibani[1,2], Luca Capua[1], Sadegh Kamaei[1], Sayedeh Shirin Afyouni Akbari[3] , Junrui Zhang[4], Hoel Guerin[4], Adrian M. Ionescu[1#]

[1]Nanolab, Ecole Polytechnique Fédérale de Lausanne, Lausanne, Switzerland

[2]Center of Excellence in Electrochemistry, School of Chemistry, University of Tehran, Tehran, Iran

[3]Advanced NEMS Laboratory, Ecole Polytechnique Fédérale de Lausanne, Lausanne, Switzerland

[4]Xsensio SA, Lausanne, Switzerland

#corresponding author: adrian.ionescu@epfl.ch

Supplementary Note 1. Chemicals and reagents

NaCl, $MgCl_2$, Ammonium persulphate, Dimethylformamide (DMF), Isopropanol (IPA), Methanol, Acetone, Poly(methy methacrylate) (PMMA) 950 K and Thris-Hcl buffer solution (1 M pH: 7.5) were obtained from Sigma Aldrich with analytical grade. PBS 1x buffer was purchased from Biochrom GmbH and 1-Pyrenebutyric acid N-hydroxysuccinimide ester (PBSE) was purchased from Lumiprobe. Hydrocortisone and testosterone were obtained from Abcr GmBH and Applichem GmbH, respectively. Chemical Vapor Deposition (CVD) grown monolayer graphene on copper substrate (Cu) was prepared by Graphenea. Cortisol aptamer has 61 nucleotides and was synthesized by Mycrosynth with 5′-amine modification. The final sequence is 5′-amine-AG CAG CAC AGA GGT CAGATG CAA ACC ACA CCT GAG TGG TTAGCG TAT GTC ATT TAC GGACC[2].

Supplementary Note 2. Configuration of the Pt electrode

Sputtered Pt electrodes on glass substrate were fabricated by MicruX Technologies. Each chip has a central circle printed Pt electrode with a thickness of 150 nm and the diameter of 1 mm. After the specific functionalization with graphene and aptamers described in the following sections, each electrode was connected to the gate of an n-channel MOSFET through a customized PCB for the realization of the extended gate cortisol sensor.

Supplementary Note 3. Graphene transfer on the Pt electrode

A layer of PMMA 950 k was spin-coated (3000 rpm for 60 s) on to the top side of the Cu/Graphene. Then the Cu/Graphene covered by the carrier polymer was baked on the hotplate at 180^0C for 2 minutes. Then, the backside graphene was etched by oxygen plasma with the power of 50 W and 6 sccm flow rate for 2 minutes. Next, a 5mm x 5mm piece was cut from the big one and floated in 0.1 M Ammonium persulfate solution for 2 hours to dissolve the copper substrate. After this step, the Ammonium persulfate solution was gently substituted with DI water by dropper. This substitution process was repeated for 3 times and finally the floated PMMA/graphene was left in DI water for 24 h to remove residuals from the graphene sheet. Graphene was then fished out onto the central Pt electrode on the chip. It should be noted that the Pt electrode surface was treated by oxygen plasma before the graphene transfer process to improve the adhesion between Pt electrode and graphene sheet. For this purpose, the chip was placed in the oxygen plasma chamber with the power of 200 W and the flow rate of 200 sccm for 30 seconds. At the end, the chip was placed in an acetone bath for 6 hours to remove PMMA. Then, it was rinsed with IPA and dried with N_2 gas gently. The last two steps were done carefully to have an intact graphene sheet. After transfer of the Graphene, the following functionalization steps were done which are described in the next part. The Schematic of different steps for graphene transfer and the last functionalized electrode with aptamers is depicted in Fig. S1.

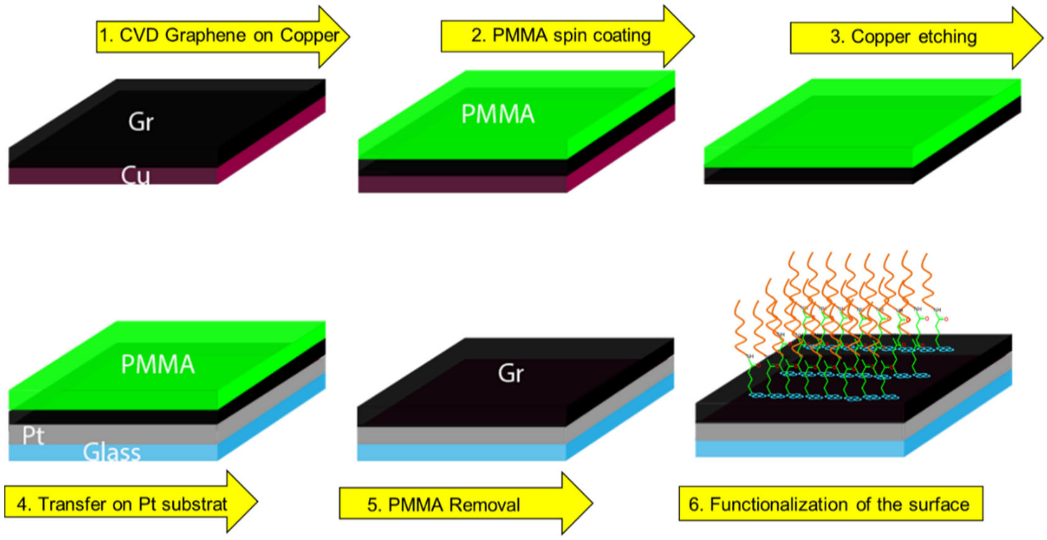

Fig. S1 Schematic of different steps carried out for graphene transfer on the surface of Pt electrode and the last functionalized electrode with aptamers.

Supplementary Note 4. EIS experiment

Electrochemical impedance spectroscopic (EIS) measurements were performed by a PalmSens electrochemical workstation (PalmSens Instruments BV, The Netherlands). This experiment was carried out in 0.05X PBS buffer solution (pH: 7.4) containing 5 mM Fe^{3+}/Fe^{4+} with a conventional three-electrode system. In this system the platinum and Ag/AgCl electrodes act as a counter and reference electrodes respectively, while the functionalized electrode is the working electrode. Impedance spectra were measured in the frequency range of 0.05–10^6 Hz. The sinusoidal voltage with the amplitude of 10 mV was applied in each step. The impedance data were fitted with a Randles circuit model to extract the corresponding charge transfer resistance.

Supplementary Note 5. AFM experiment

Topography analysis of the electrodes were carried out with a Cypher Atomic force microscope (Asylum Research) in non-contact mode. AFM imaging was done for the Pt/graphene electrode and the following functionalization steps of the surface. In each step before imaging, the surface of the electrode was rinsed several times and dried with N_2 gas.

Supplementary Note 6. XPS experiment

To elaborate the chemical functionalization of the electrode surface, X-ray photoelectron spectroscopy (XPS) analysis was utilized in different steps of electrode functionalization. XPS analysis was carried out using a PHI Versa Probe II scanning XPS microprobe (Physical Instruments AG, Germany) to characterize the representative graphene surfaces before and after functionalization with PBSE and simulated aptamer probes. A simulated probe sequence containing 62 thymine nucleotides (T_{62}) was utilized instead of the cortisol aptamer sequence to produce an XPS signature. This is due to the fact that the interpretation of the data would be easier in terms of density and conformation of the surface-immobilized nucleic acid strands[3,4]. Analysis was performed using a monochromatic Al Kα X-ray source of 24.8 W power with a beam size of 100 µm. The spherical capacitor analyzer was set at 45° take-off angle with respect to the sample surface. The pass energy was 46.95 eV yielding a full width at half maximum of 0.91 eV for the Ag 3d 5/2 peak.

The C1s peak located at 284.8 eV for all the samples. In addition, the high resolution of Pt 4f region contains a pairs of peaks for all the measured samples (Pt 4f7/2 and Pt 4f5/2) which is a characteristic of metallic Pt (0) with the binding energy of 71.3 and 74.5 eV, respectively.

Supplementary Note 7. Experimental set up for EGFET sensor characterization

The characterizations of the modified EGFET was investigated by semiconductor parameter analyzer HP 4155B. The chip containing modified electrode was placed on the base of a static cell (purchased from MicruX Technologies). When the cap of the cell is placed on top of the chip, an open static cell with volume of the 400 µl is formed. The modified electrode was connected via pin to the gate of the commercial n-channel 0.18 µm MOSFET, which was placed on a printed circuit board (PCB). The connections of the source and drain were designed on the PCB to be connected to the parameter analyzer. The gate voltage was applied to an Ag/AgCl reference electrode, which was placed in the solution of the static cell.

Supplementary Note 8. Confirmation of the aptamer functionalization by recording EGFET transfer characteristic

In order to confirm the correct immobilization of the aptamers on the graphene surface, the transfer characteristic of an EGFET sensor using a larger size transistor was recorded in the measurement solution (PBS buffer 0.05X pH: 7.4) after the immobilization of the aptamers, and after exposure of the functionalized electrode to the lowest tested concentration of the cortisol (0.2 nM). As shown in Supplementary Fig. S2, both the immobilization of the aptamers on the graphene surface and the exposure of the functionalized electrode to the cortisol solution, lead to right shift in the I_{DS}-(V_{REF}-V_T) curves. In Fig. S2, the drain current is plotted against the difference between the applied reference voltage and the threshold voltage, which was extracted from the data with the second derivative of the drain current method, a method that is independent of the model adopted for the transfer characteristics. Aptamers are negatively charged molecules introduced to the surface of the electrode, also binding of cortisol to the aptamers makes the strands to fold on themselves, and come closer to the surface[5]. Consequently, the surface potential of the electrode, ψ, is modulated in the steps of electrode functionalization with aptamers and exposure of the functionalized electrode to the cortisol. Due to the relation existing between the threshold voltage, V_T, and the surface potential, ψ (as described in the main article), the V_T of the EGFET is modulated according

to the amount of negative charges induced by the aptamers. Therefore, a higher gate voltage is required to obtain the same drain current, which results in a right shift of the I_{DS}-(V_{REF}-V_T) curves.

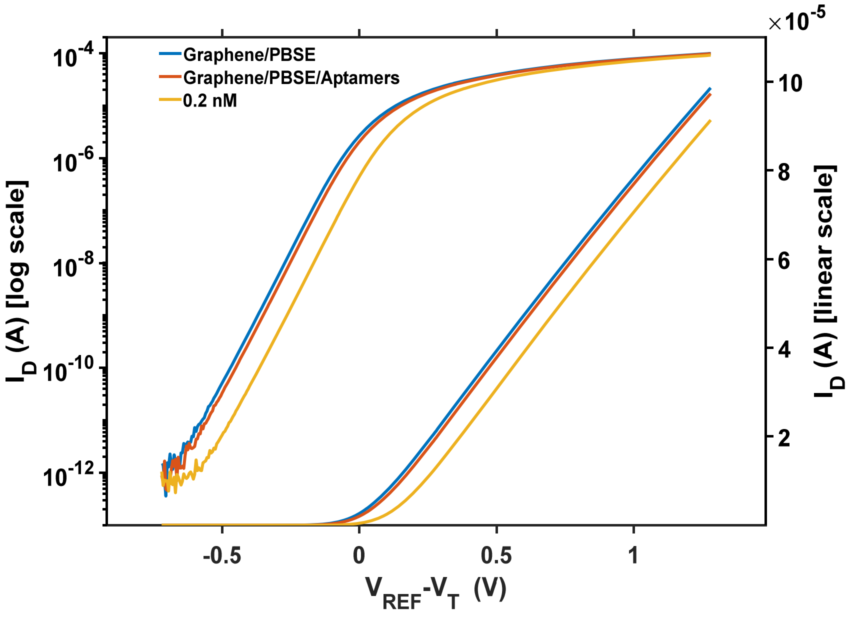

Fig. S2 Current-Voltage response of the sensor towards functionalization steps. The steps include the attachment of the aptamers on the surface of the Pt/graphene electrode and then binding of the cortisol with the amount of 0.2 nM to the functionalized surface of the electrode (Pt/graphene/aptamer).

Supplementary Note 9. Analyze of the low concentrations of the cortisol with the sensor

For investigation the resolution of the sensor in low concentration range, the sensor was exposed to different concentrations of the cortisol between 1 and 100 nM and the response of the sensor was recorded. Fig. S3a shows that the transfer characteristics, ID-(V_{REF}-V_T), shift to *right* axis direction with increasing the concentration of the cortisol. In Supplementary Fig. S3b the sensitivity of the cortisol sensor in the low concentration range is extracted from the slope of V_{REF} versus concentration showing remarkably close values (15.7 to 16. 9mV/decade) to the ones reported in the main paper on the whole range of 4 orders of magnitude of varying concentration at constants current for these concentrations. The family of curves in Fig. S3b corresponds to various extractions made at constant current levels from weak to strong inversion, all showing similar sensitivities.

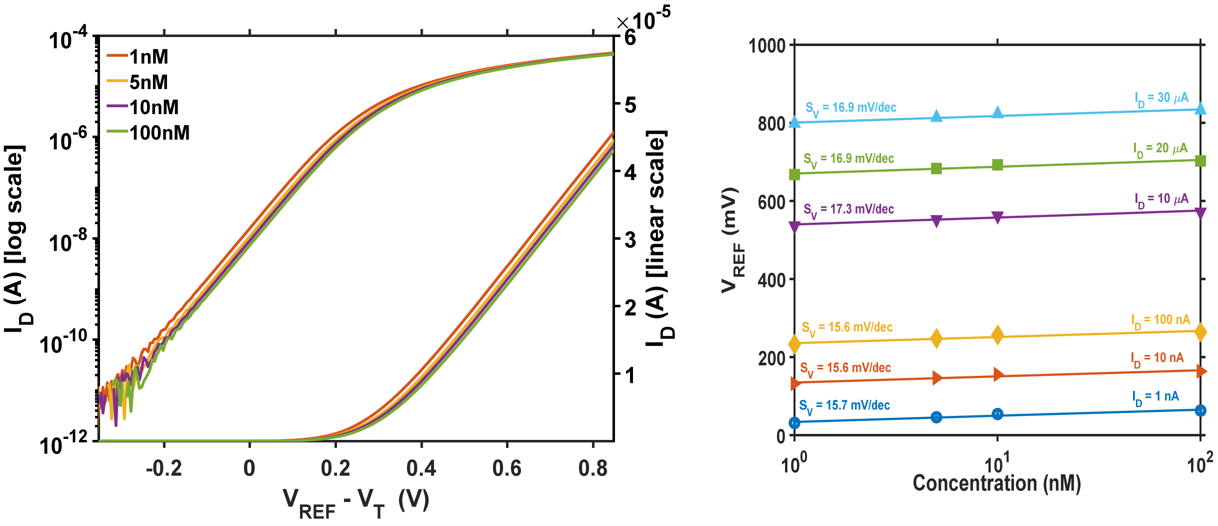

(a) (b)

Fig. S3 Current-Voltage plots and sensitivity on cortisol concentration of EG-FET in the 1-100nM range. a) I_D-(V_{REF}-V_T) transfer characteristics of cortisol sensor, in semi-logarithmic and linear scale for different cortisol concentrations. b) Voltage sensitivity, S_V, for different current levels from weak to strong inversion operation.

Supplementary Note 10. Unified modeling: fitting, parameter extraction and accuracy

In this section we describe the procedure used to calibrate the unified model of Equation (2) reported in the main paper, the values of the model parameters used in the paper and the model accuracy obtained versus experimental data. Moreover, we report the calibration of the analytic dependences of V_T on concentration, using experimental data for every cortisol concentration.

From a physical point of view, the unified Equation (2) of the main paper has as physical parameters, the threshold voltage, V_T, the transistor body factor, η , the temperature of operation, T and the specific transistor geometry factor, $K_n = \frac{W}{L}\mu_0 C_{ox}$, where W/L is the channel width over length ratio, μ_0 is the low-field mobility, and C_{ox} is the gate oxide capacitance. It is worth noting that this equation can be mathematically simplified into the traditional well-known drain current equations in weak (for V_G-$V_T < 0$) and strong inversion (for $exp((V_{GS}-V_T)/nU_T) >> 1$), respectively:

$$I_D(V_{GS}) \sim (W/L)\, I_M e^{\left(\frac{(V_{GS}-V_T)}{\eta U_T}\right)} \left(1 - e^{\frac{-V_{DS}}{U_T}}\right) \quad \text{(S1)}$$

$$I_D(V_{GS}) \sim (W/L)\mu_{eff}\, C_{ox}(V_{GS} - V_T)V_{DS} \quad \text{(S2)}$$

as used by many authors in the literature. For the sensor transfer characteristics, I_D-V_{GS}, the unified Equation (2) has been fitted on the whole experimental data with a standard non-linear least

squares error minimization algorithm in Python, providing r^2=0.98 after fitting at the lowest cortisol concentration, considered as a reference, resulting in the following values: V_{T0} = 0.0859 V, K_n = 2.0 10^{-3}A V^{-2} , η = 1.4. A dedicated extraction method, called the Y function[5], using both drain current and transconductance versus gate voltage, and offering the best accuracy for the extraction of the threshold voltage, V_{T0}, has been used to double check the physical coherence of values obtained with the fitting procedure. It should be also noted that we have directly extracted the value of K_n and not independently the low field mobility and gate capacitance, for the existing device. Data in this paper correspond to a large EG-FET device, having W=22.6µm and L= 21.6µm, this W/L = 1.046.

For calibrating the unified Equation (3), with the explicit dependence of the drain current on the cortisol concentration, we have used a logarithmic dependence of the MOSFET threshold voltage, V_T, on the cortisol concentration:

$$V_T = V_{T0} + \Delta V_{T0} + m \ln\left(\frac{C+C_{corr}}{C_{REF}}\right) \quad \text{(S3)}$$

Equation (S3) has been fitted on the extracted V_T data for the applied concentration of cortisol (10, 100 and 10'000 nM), resulting in an excellent agreement between experiments and model, as seen in Fig. S4b. The parameters of Equation (S3) are the threshold voltage, V_{T0}, value for C_{REF0}=1nM (their value are reported in Fig. S4b caption). It should be noted that when calibrating the model on data, we observed a possible imprecision on the lowest value of 1nM of cortisol concentration in our solution, which motivated the introduction of the parameters ΔV_{T0} and C_{corr} in the model, to obtain the best fit over all our experimental data, from 1nM to 10'000nM. The fitting suggest that the lowest reference concentration should be corrected to 7nM instead of 1nM, with C_{corr} = 6nM, leading to $\Delta V_{T0} = -0.0157$ V. While the preparation of our cortisol buffers has been extremely careful and precise, such suggested model parameter correction for the nanomolar range can be related to two reasons: (i) the limitations of the proposed model to capture some specific change in the response of the EG-FET sensor (and sensitivity) due to Langmuir adsorption effect in the very low concentration range, and, (ii) the higher influence of experimental conditions in the low concentration rage due to the microfluidic setup.

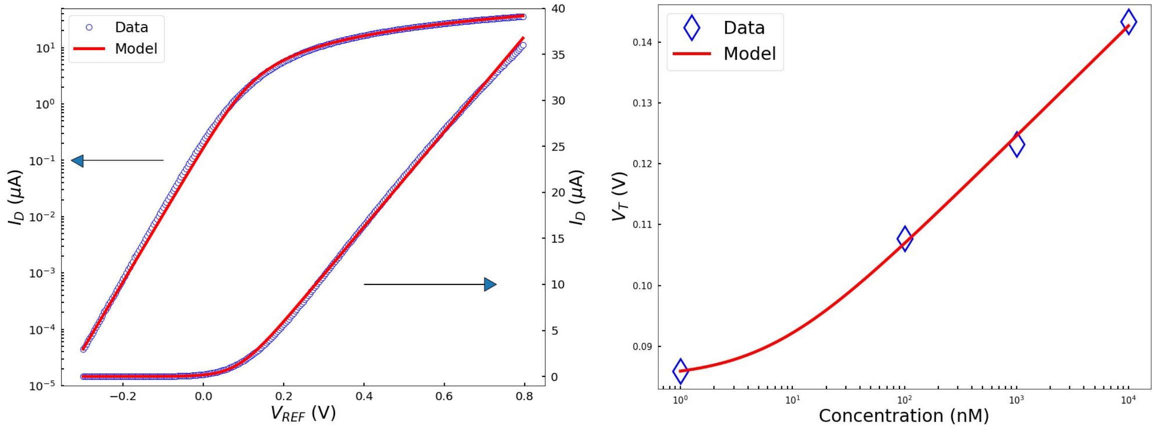

(a) (b)

Fig. S4 Comparison of the unified model (solid lines) with measurements (symbols). Transfer characteristics, I_D - V_{REF}, in log-lin and lin-lin scales, for the lowest cortisol concentration, using the following extracted parameters: η = 1.4, V_{T0} = 0.0859 V, and K_n = 2.0 · 10^{-3}A V^{-2}.b) Threshold voltages for each cortisol concentration modeled by equation (6) with m = 0.00787V, $\Delta V_{T0} = -0.0157$ V, and C_{corr} = 6 nM, versus extracted experimental threshold voltages at constant current. The models of Equation (2) and Equation (S3) show very good accuracy.

The calibrated threshold voltage dependence on concentration of Fig S4b, is used in the unified closed-form drain current Equation (3) of the paper to predict the drain current of the cortisol EGFET sensor at every bias point and at every cortisol concentration; the excellent accuracy of the model is shown in Fig. S5 that depicts the full I_D-V_{GS} EG-FET characteristics, from weak to strong inversion, at the four investigated cortisol concentrations, with the model parameters extracted in Figs. S4a-b.

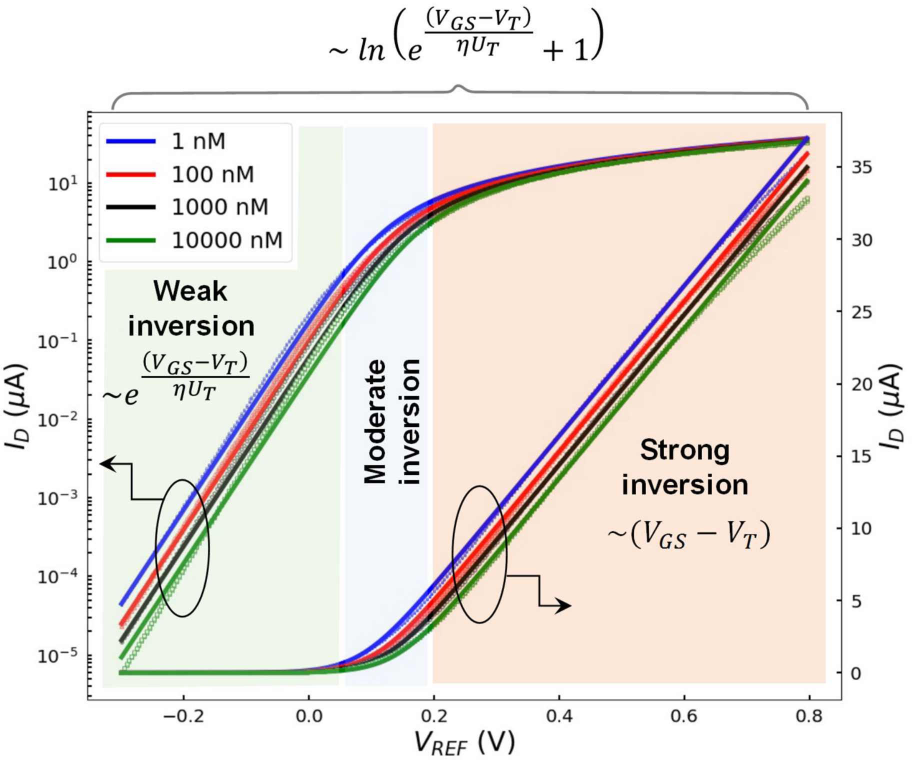

Fig. S5 Unified model versus experimental I-V experimental data of cortisol EG-FET. Comparison of the unified model (solid lines) based on Equation (3) of the main paper, with measurements (symbols): transfer characteristics, I_D - V_{REF}, in *log* - *lin* and *lin* - *lin* scales in all regimes of operation, demonstrating an excellent accuracy at every bias and cortisol concentration.

Supplementary Note 11. Study of the selectivity

In this work, testosterone and cortisone were chosen for the investigation of the cortisol sensor selectivity. Testosterone and cortisol are adrenal hormones and have similar structures which may raise the question of the cross-sensitivity of the aptamer functionalization to testosterone. The amount (concentration) of the testosterone in biofluids such as blood and sweat, is much lower than the cortisol. For instance, the level of testosterone in blood ranges from 0.52 to 2.4 nM for females and from 9.4 to 37 nM, for males. Cortisone in turn metabolized from cortisol in the peripheral tissues and has the most similar structure to the cortisol. However, cortisone has much less glucocorticoid activity than cortisol, so it can be considered an inactive metabolite of cortisol[6].

The fabricated cortisol sensor was exposed to testosterone in the range of the concentrations similar to testosterone concentrations in biofluids. Also the sensor was exposed to the cortisone in the range of the concentrations similar to the cortisol measurement. The stock solutions of the

testosterone and cortisone with the concentration of 0.01 M was made in methanol, then all the other diluted solutions were prepared from the stock solution in the incubation buffer used for cortisol measurements. The tests were carried out in the same conditions as for cortisol. As can be seen from Fig. S6a, when the sensor is exposed to the lowest concentration of the testosterone (10 pM), the corresponding I_{DS}-(V_{REF}-V_T) curve shifts to the left direction compared to the characteristic of the sensor in a buffer with no testosterone. As the testosterone concentration increases in successive steps (10 pM, 100 pM, 1 nM) up to 10 nM, the I_{DS}-V_{REF} curves show no significant trend and tend to overlap. For testosterone only a very slight shift in the left direction was observed, with no significant trend. The results for the sensor response to the cortisone is depicted in Supplementary Fig. S6b, as can be seen when the sensor is exposed to the lowest concentration of the cortisone (1 nM), the corresponding I_{DS}-(V_{REF}-V_T) curve shifts to the right direction compared to the characteristic of the sensor in a buffer with no cortisone. However, as the cortisone concentration increases up to 10 μM, the I_{DS}-V_{REF} curves overlap, and no trend was observed. These results for testosterone and cortisone are in contrast to the clear sensor response to cortisol (for which all curves are shifting systematically to the right when the cortisol

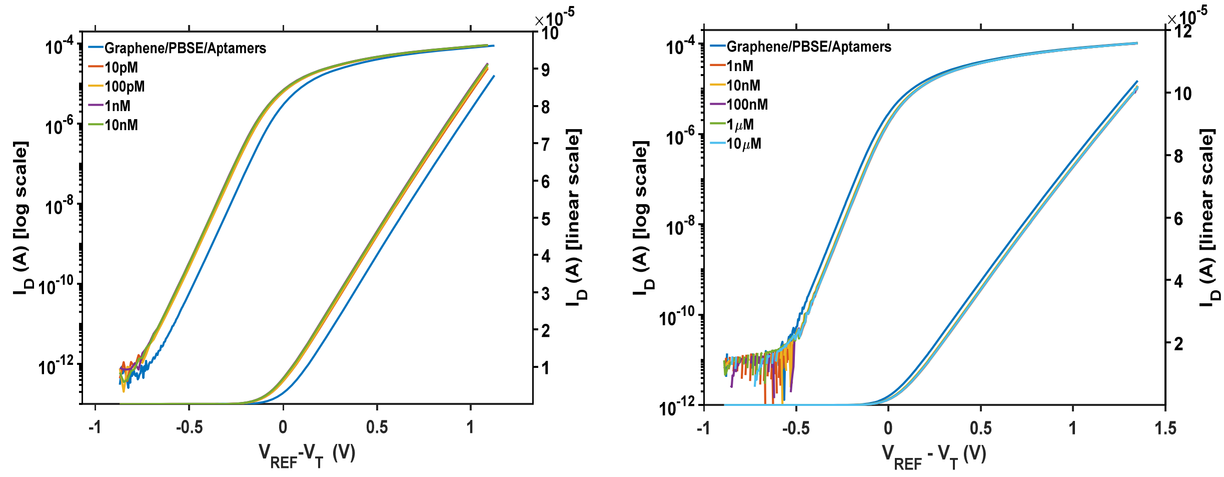

(a) (b)

concentration increases).

Fig. S6 Experimental verification of EG-FET sensor selectivity versus testosterone and cortisone. I_D-V_G response of the sensor towards different concentrations of (a) testosterone and (b) cortisone as an interfering biomolecules.

In addition to the data reported in Fig. S6, the shift of the I_{DS}-V_{REF} characteristics in terms of V_{REF} voltage at constant current of 1 μA was measured under the presence of cortisol alone and under the combination of both cortisol and testosterone or cortisone. When the sensor was exposed to the lowest concentration of cortisol (with no testosterone), a positive shift to the right, of 80 mV for a concentration of 0.2 nM of cortisol corresponding to the LOD, was recorded. This shift was reduced to a value of 70 mV when the highest tested concentration of testosterone (10 nM) has been added to the same solution. However, when the highest concentration of the cortisone (10 μM) was added to the solution containing lowest tested concentration of the cortisol, almost no shift of I_{DS}-V_{REF} curve was observed. Therefore, it is concluded that our sensor is highly selective to cortisol and only its LOD can be slightly increased in the presence of highest levels of testosterone in human biofluids. Further experiments with very low concentrations of cortisol (below 1nM) and high concentrations of testosterone are needed to be able to quantify such extremely small cross-sensitivity.

Supplementary Note 12. Study of the drift effect

It is well-known that ISFET sensors could exhibit an instability, commonly known as drift, in the form of a slow, monotonic, temporal increase in the threshold voltage of the device. The study of such drift effect is important to estimate any potential influence of the experimental data used for the sensitivity evaluation. Moreover, the precise knowledge on the drift is very useful as it can be used to correct the sensing recorded data and offer an accurate estimate of the real sensor response. In order to study the drift, we have designed a multi-step experiment: (i) first, the response of the freshly functionalized sensor was recorded, (ii) then the sensor was kept in an incubation buffer solution without cortisol for 30 minutes, which is the time required for the cortisol binding, (iii) afterwards, the sensing electrode was rinsed and placed in the same buffer used for the cortisol measurement and the transfer characteristics, I_D-V_{GS}, of the EGFET were recorded. This three-step process was repeated for three consecutive times.

As shown in Fig. S7, the shift between I_D-(V_{REF}-V_T) curves for the mentioned experiments is quasi-negligible and after 90 minutes, the sensor shows a stable response. We conclude that any potential drift influence is minor for our sensor and our experiments with the aptamer functionalized graphene/Pt electrode electrically connected to the gate of the FET.

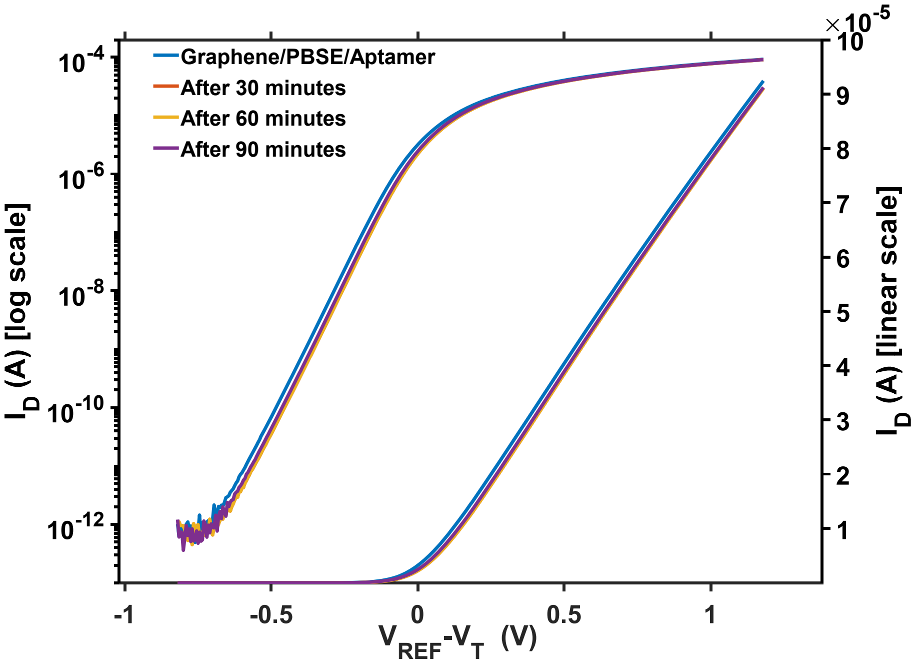

Fig. S7 Drift study of the proposed EG_FET. I_D-V_G response of the sensor after functionalization with the aptamers and after exposing the sensor into an incubation buffer for 30 minutes for 3 consecutive times.

Supplementary References

1. Chen, R. J., Zhang, Y., Wang, D. & Dai, H. Noncovalent Sidewall Functionalization of Single-Walled Carbon Nanotubes for Protein Immobilization. *J. Am. Chem. Soc.* **123**, 3838–3839 (2001).
2. Fernandez, R. E. *et al.* Disposable aptamer-sensor aided by magnetic nanoparticle enrichment for detection of salivary cortisol variations in obstructive sleep apnea patients. *Sci. Rep.* **7**, 1–9 (2017).
3. Schreiner, S. M. *et al.* Controlled and Efficient Hybridization Achieved with DNA Probes Immobilized Solely through Preferential DNA-Substrate Interactions. *Anal. Chem.* **82**, 2803–2810 (2010).
4. Petrovykh, D. Y., Kimura-Suda, H., Tarlov, M. J. & Whitman, L. J. Quantitative Characterization of DNA Films by X-ray Photoelectron Spectroscopy. *Langmuir* **20**, 429–440 (2004).
5. Xu, X. *et al.* Reconfigurable carbon nanotube multiplexed sensing devices. *Nano Lett.* **18**, 4130–4135 (2018).

6. Rippe, R. C. A. *et al.* Splitting hair for cortisol? Associations of socio-economic status, ethnicity, hair color, gender and other child characteristics with hair cortisol and cortisone. *Psychoneuroendocrinology* **66**, 56–64 (2016).
